# Vitamin D in Vascular Calcification: A Double-Edged Sword?

**DOI:** 10.3390/nu10050652

**Published:** 2018-05-22

**Authors:** Jeffrey Wang, Jimmy J. Zhou, Graham R. Robertson, Vincent W. Lee

**Affiliations:** 1 Centre for Transplantation and Renal Research, Westmead Institute of Medical Research, Westmead, NSW 2145, Australia; jwan3534@uni.sydney.edu.au (J.W.); jimmy.zhou@health.nsw.gov.au (J.J.Z.); 2 Centre for Kidney Research, Children’s Hospital at Westmead, Westmead, NSW 2145, Australia; 3 Cellmid Limited, 2/55 Clarence St, Sydney, NSW 2000, Australia; robertson@cellmid.com.au

**Keywords:** vascular calcification, osteogenic differentiation, calcium, phosphate, vitamin D, hypervitaminosis, hypovitaminosis, biphasic

## Abstract

Vascular calcification (VC) as a manifestation of perturbed mineral balance, is associated with aging, diabetes and kidney dysfunction, as well as poorer patient outcomes. Due to the current limited understanding of the pathophysiology of vascular calcification, the development of effective preventative and therapeutic strategies remains a significant clinical challenge. Recent evidence suggests that traditional risk factors for cardiovascular disease, such as left ventricular hypertrophy and dyslipidaemia, fail to account for clinical observations of vascular calcification. Therefore, more complex underlying processes involving physiochemical changes to mineral balance, vascular remodelling and perturbed hormonal responses such as parathyroid hormone (PTH) and fibroblast growth factor 23 (FGF-23) are likely to contribute to VC. In particular, VC resulting from modifications to calcium, phosphate and vitamin D homeostasis has been recently elucidated. Notably, deregulation of vitamin D metabolism, dietary calcium intake and renal mineral handling are associated with imbalances in systemic calcium and phosphate levels and endothelial cell dysfunction, which can modulate both bone and soft tissue calcification. This review addresses the current understanding of VC pathophysiology, with a focus on the pathogenic role of vitamin D that has provided new insights into the mechanisms of VC.

## 1. Introduction

Vascular calcification (VC) is a complex disorder of both major and minor blood vessels that is primarily characterised by calcium deposition along vascular walls. These deposits predominantly consist of calcium and phosphate minerals in the form of hydroxyapatite crystals, which incidentally are the same mineral components found in bone [1,2]. While mineralisation of calcium and phosphate is necessary for normal bone development and turnover, occurrences beyond bone tissue are generally pathological, and regarded as extraosseous calcification [3,4]. Within blood vessels, expansion of calcification typically leads to vessel stiffening and reduced compliance, which explains why VC is highly associated with cardiovascular mortality. VC is commonly found within the chronic kidney disease (CKD) population and its prevalence increases with CKD progression [5,6,7]. Indeed, up to 3–4 fold increase in VC has been reported in CKD patients during the early development of progressive renal injury and dysfunction [8]. Ectopic calcification can typically occur within the intimal and medial layers of major peripheral arteries [9,10] (referred to as intimal and medial calcification respectively), as well as the myocardium, cardiac valves and coronary arteries (referred to as coronary artery calcification) [11,12]. A rare and obliterative form of VC known as calcific uremic arteriolopathy, or calciphylaxis, occurs almost exclusively within end-stage renal disease (ESRD) patients, and is characterised by extensive skin necrosis, ulcer formation and visceral plaque accumulation [13,14,15]. Regardless of the type, CKD patients with manifestations of extraosseous calcification are predisposed to cardiovascular events, such as worsening ventricular hypertrophy, myocardial infarction, arrhythmia and strokes, and generally have poorer prognosis and increased mortality rate, which is on average 20–30 times higher as compared to the general population [16,17,18,19,20]. Despite its clinical relevance, therapeutic measures designed to manipulate the different risk factors of VC still fail to demonstrate a significant impact on patient survival, which may be largely due to the lack of understanding of VC pathophysiology. For some time VC was thought to be a clear manifestation of atherosclerosis, and strongly associated with diabetes, hypertension and dyslipidaemia [21,22,23,24,25]. However, recent studies show that these risk factors fail to explain the abnormal VC dimensions and outcomes that persist in long-term patients, suggesting an additional role for non-traditional risk factors including hormonal perturbations, cellular modifications and disordered mineral metabolism. 

## 2. Pathophysiology of VC

The theories behind VC have continued to evolve since the first characterisation of calcified vessels. Historically, based on the high calcium and phosphate content derived from these vessels, VC was described as a passive and degenerative process of calcium and phosphate accumulation [26]. Following the recognition of additional extraosseous bone-like mineralised tissue within these vessels, VC was subsequently acknowledged as an active, pathobiological process with some features of bone morphogenesis, but also displaying distinct cellular and molecular processes. A recent study emphasised this concept by demonstrating the propensity of VC to persist despite correcting for mineral imbalance within an adenine-induced advanced CKD animal model [27]. In this study, aortic calcification was exacerbated in CKD-induced mice by supplementation of a high-phosphate diet and periodic administration of calcitriol (1,25-dihydroxyvitamin D3). Lomashvili et al. were then able to elegantly demonstrate that upon dissecting and transplanting segments of calcified aorta into healthy littermates, moderate decreases in aortic calcium content could be initially observed, but calcified tissue persisted and remained intact after a 35 week follow-up period [27]. Furthermore, calcified vessels continued to mineralise with calcium-phosphate deposition similar to that of hydroxyapatite in bone. These findings therefore revealed that even if perturbed systemic levels of calcium and phosphate are restored, calcification can still persevere indicating that it is an actively instigated and regulated process.

Although the underlying mechanism(s) are complex and still remain to be fully elucidated, the scientific literature has alluded to ossification as one of the principle active processes which orchestrates VC. Indeed, this idea is supported by the prevalence of regulators of bone formation and bone structural proteins such as bone morphogenic protein (BMP) and osteopontin (OPN) within calcified vascular tissue [28,29,30]. Further evidence to reinforce this concept also comes from studies concerning kidney calcification, which pathologically manifests as stone formation in tubular microenvironments, such as the kidney tubules [31]. The resemblance in the biomineralisation process leading to kidney stone formation to that of VC suggests that bone regulators that have been documented to mediate stone formation, are therefore very likely to propagate VC as well. Given the presence of bone elements, it is therefore important to recognise calcified vasculature as manifestations of bone-like tissue encapsulating osteoblast-like cells and haematopoietic elements, with calcium and phosphate actively accumulating as part of the bone matrix composition rather than stagnant mineral deposits. There is substantial clinical and experimental evidence suggesting that osteoblast-like cells within calcified tissue can originate from pericytes in microvessels, pericyte-like vascular and endothelial cells in the intima, and vascular smooth muscle cells (VSMCs) in the media which undergo osteogenic/chondrogenic differentiation in response to osteogenic signals. These cells, much like osteoblasts and osteocytes themselves, express bone-related markers and structural proteins, and engage in ossification through the secretion of matrix vesicles and mineralisation of calcium and phosphate in the form of hydroxyapatite [32,33,34,35,36]. Results from in vitro studies of vascular cell calcification have shown that primary cultures of VSMCs can be induced to express osteogenic markers, such as Runt-related transcription factor 2 (RUNX2), following treatment with the inorganic phosphate donor, beta glycerophosphate [37]. Additionally, these cells also lose their expression of smooth-muscle specific markers, such as SM22 alpha, and hence undergo phenotypic switching. Similarly, endothelial dysfunction through stress or trauma may promote endothelial cells to undergo endothelial-mesenchymal transition (EMT), which subsequently generates multipotent osteoprogenitor cells that, much like de-differentiated VSMCs, transform into osteogenic cells capable of mineralisation. Important molecular regulators involved in this process have been identified, most notably BMPs that propagate the process, as well as the matrix Gla protein (MGP) that is a critical regulator of BMP activity. Experimental studies have shown that increased MGP expression is associated with reduced BMP and EMT activity, and subsequently calcification outcomes [38,39]. In addition, endothelial dysfunction may promote VSMC osteogenic differentiation through crosstalk between the endothelial and medial layers, therefore furthering calcification outcomes. Interestingly, clinical evidence indicates that current efforts aimed at reversing the extent of ossification in all vascular branches are insufficient in ameliorating the progression of calcification and arterial stiffness, suggesting that the process is more complex with other aspects besides regulators of bone formation to consider in its pathogenesis [26]. 

## 3. Risk Factors for VC

As VC frequently occurs with atherosclerotic and arteriosclerotic diseases, initial thoughts on its risk factors were centred on cardiovascular outcomes. Indeed, early studies of VC focused on its development in association with traditional metabolic risk factors such as age, smoking, alcohol consumption and measures of adiposity [22]. Clinically, patients with diabetes, dyslipidaemia or hypertension frequently develop VC, which is suspected to augment susceptibility to cardiovascular-related events [40,41]. As such, early interventions for VC were predicated on correcting general metabolic disorders that compromised cardiovascular health. The primary limitation that clinicians currently face in considering these traditional risk factors for VC treatment is that they fail to account for a large number of long-term VC cases, particularly those which manifest in CKD populations. In many of these cases, the severity of calcification correlates with the progressive loss of renal function and CKD stage independently of atherosclerotic or arteriosclerotic outcomes [7,42]. VC has also been shown to increase the risk of mortality within these patients above that predicted by traditional cardiovascular risk factors. For example, Chen et al. recently concluded that after a follow-up of 6 years, patient coronary artery calcification (CAC) scores were independently associated with composite cardiovascular outcomes after correcting for novel and traditional cardiovascular risk factors [43]. The addition of CAC scores also improved upon the prediction model for CKD prognosis, which at the time only included conventional factors such as baseline estimated glomerular filtration rate (eGFR) and proteinuria. One of the major reasons why CKD patients may display less predictable VC patterns lies in the fact that more physiological systems become deregulated within the renal-skeletal-cardiovascular axis under CKD conditions [44,45]. Due to the complexity of its pathophysiology within the CKD population, new studies have emerged examining the role of various non-traditional risk factors in VC development. 

### 3.1. Calcification Promoters and Inhibitors

Recent evidence indicates that many non-atherosclerotic calcification cases may be a result of modulations in the levels and activities of calcification promoters and inhibitors. Indeed, VSMCs readily secrete such mediators of calcium regulation into their extracellular environment during vascular remodelling, and it has been hypothesised that calcification ensues following net changes in promoter and inhibitor levels respectively. Alkaline phosphatase for example is a major matrix vesicle constituent secreted by de-differentiated VSMCs, that promotes calcification through the degradation of pyrophosphate [46]. Clinical and experimental studies have demonstrated that pyrophosphate deficiency resulting from alkaline phosphatase activity during vascular remodelling promptly stimulates biomineralisation [47,48]. As for calcification inhibitors, examples include Fetuin-A and MGP which have been found in blood vessels, and a lack of these inhibitors has been linked with spontaneous calcification and increased mortality [49,50]. Although these molecular regulators modulate different pathways, they commonly invoke smooth muscle osteogenic differentiation and mineralisation as primary calcification mechanisms, with promoters upregulating and inhibitors downregulating these processes respectively.

### 3.2. Calcium and Phosphate

There is also growing evidence indicating that within a large number of VC cases, physiochemical changes to mineral metabolism actively contribute, rather than being a passive outcome of its pathophysiology. Of relevance, perturbed levels of calcium and phosphate have been shown to mediate VC pathogenesis and development [51]. Traditionally, calcium and phosphate were thought to drive calcification thermodynamically, whereby saturated levels eventually lead to passive precipitation of calcified deposits. While this may be the case in normal chemical solutions, in the vasculature, calcium and phosphate levels are tightly regulated under physiological conditions such that spontaneous accumulation is less likely to induce calcification [52]. Furthermore, as the concepts around calcification regulation have evolved to become more complex, it is unlikely that the primary mechanism responsible for its pathogenesis and progression would involve such a passive and linear process. New evidence suggests that both calcium and phosphate may have a more active function in VC by regulating VSMCs, and predisposing them to transform into bone-like tissue. Studies have shown that phosphate can directly drive the osteogenic differentiation and mineralisation of VSMCs, while calcium indirectly promotes these mechanisms by primarily modulating phosphate activity [53].

In vitro manipulation of calcium and phosphate levels in culture media of VSMCs to hypercalcaemic and hyperphosphatemic conditions respectively leads to the loss of smooth muscle cell markers and upregulation of genes commonly associated with bone formation [54,55]. Notably, elevated extracellular phosphate levels stimulate the expression of the sodium-dependent phosphate transporter Pit-1 on the cell surface of VSMCs, thereby promoting phosphate uptake [56]. Consequently, increased intracellular phosphate directly induces the expression of osteogenic genes, such as RUNX2 and osteopontin [57,58]. Elevated calcium levels on the other hand have been shown to augment Pit-1 expression, and therefore indirectly contribute to smooth muscle osteogenesis by enhancing phosphate uptake [59,60]. Whether calcium itself can directly signal the osteogenic differentiation pathway of VSMCs has yet to be established. 

Concomitant with VSMC phenotypic switching, increased calcium and phosphate loading also promotes mineralisation through stimulating the secretion of matrix vesicles and apoptotic bodies, which become the primary nucleation sites for calcification through the de novo synthesis of hydroxyapatite crystals [58,60,61,62]. Passive deposition of excess calcium and phosphate would also encourage the growth of these crystals, thereby promoting their expansion. Consistent with these in vitro findings, similar observations have also been made using ex vivo human samples, as well as in vivo animal models of VC, where free serum calcium and phosphate levels were linked to ossification of soft tissue [53,63,64]. Clinical cases of VC have also addressed the association between calcification progression and increased serum calcium and phosphate levels, as well as calcium and phosphate regulators in patients [11,16,65,66]. 

FGF-23 in particular has recently been identified as a risk factor for VC, with several studies demonstrating correlations between elevated levels of circulating FGF-23 and increased calcification scores in CKD patients [67,68,69]. Given that the primary function of FGF-23 involves the direct and indirect regulation of phosphate and calcium levels [70,71], it therefore has the potential to augment VC outcomes in these patients by adding to the existing storm of deranged calcium and phosphate metabolism. Despite these associations, the relationship between FGF-23 and VC still remains controversial, as its levels have not been associated with VC development in patients with normal kidney function [72]. 

In summary, there is strong evidence suggesting pro-calcifying effects from both calcium and phosphate which are primarily based on their ability to actively stimulate phenotypic transformation of cells within the vasculature, including both vascular smooth muscle calcification and osteogenic matrix mineralisation. Passive accumulation on the other hand may still be a relevant mechanism that either sustains or aggravates calcification by exacerbating the mineralisation process. While most findings emphasise that calcium synergises with phosphate during the onset of calcification, whether or not these risk factors are able to independently promote the process still requires further clarification. 

## 4. The Vitamin D Hormone

Vitamin D is one of the most important steroid hormones in the body. Its predominant function is the regulation of skeletal health and mineral homeostasis through modulating calcium and phosphate metabolism at major physiological organs, including the intestines, liver, bone and kidneys. As such, its deficiency is often associated with bone-mineral disorders such as rickets and osteomalacia. Vitamin D has also been reported to serve non-skeletal functions, the majority of which are highly implicated in cardiovascular outcomes.

While it can be supplemented, vitamin D is normally derived either exogenously through dietary sources, such as fatty fish and eggs, or endogenously through synthesis in the skin. The primary source however, is through skin synthesis from exposure to ultraviolet radiation. The inactive precursor, known as cholecalciferol (vitamin D3) that is initially produced promptly undergoes 25-hydroxylation by one of four cytochrome P450 enzymes (namely vitamin D 25-hydroxylase, or CYP2R1) in the liver to produce calcifediol (25-hydroxyvitamin D), a pre-hormone generally used as the biomarker to indicate vitamin D status. Calcifediol, which is normally bound to the circulating vitamin D binding protein (DBP), can undergo a subsequent round of hydroxylation by another cytochrome P450 enzyme, 1α-hydroxylase (CYP27B) in the kidneys to produce the active vitamin D metabolite, calcitriol (1,25-dihydroxyvitamin D3), which ultimately elicits the effects of vitamin D by binding and signalling through the vitamin D receptor (VDR) in target tissues.

Perturbation of vitamin D levels can occur, with the primary manifestation being vitamin D deficiency which is predominantly caused by lack of sunlight exposure and therefore synthesis in the skin. Indeed, this point is reinforced by the increased prevalence of deficiency cases during winter and in populations further away from the equator [73,74]. Lack of synthesis of precursors in the skin primarily leads to inadequate production of calcifediol, which consequently results in reduced downstream effects. Therefore, correction of deficiency generally involves direct supplementation of calcifediol. While deficiency is widely noted, excess levels of vitamin D, or vitamin D toxicity, has also been reported and attributed to cases of kidney stones, renal impairment, and cardiovascular diseases [75]. In contrast to deficiency, toxicity has not been associated with excessive sunlight exposure, but more so with abnormal supplementation of calcifediol as over-production of inactive cholecalciferol in the skin does not result in over-production of calcifediol [76,77]. Excess supplementation of calcefidiol however, leads to saturation of free calcifediol levels that exceeds DBP binding capacity, which then directly induces gene expression and functional changes in target cells [78]. Therefore, while supplementation of calcifediol is an accepted form of treatment for vitamin deficiency, the process should be tightly monitored to ensure that levels are restored, but not overdosed to toxic levels. 

Despite the general consensus on diagnosing vitamin D-related diseases/disorders based on circulating levels, there is still controversy surrounding whether aberrant levels of vitamin D are equivalent to perturbed activity, given the nature of its metabolism and signalling. For example, production of active vitamin D (calcitriol) in the kidneys is tightly regulated through feedback mechanisms on 1α-hydroxylase activity, such as PTH and FGF-23, and therefore cannot be entirely dependent on pre-hormone levels [79]. Furthermore, circulating levels of vitamin D do not reflect its concentration in target tissues, as many tissue cells themselves express 1α-hydroxylase and are therefore able to locally regulate active vitamin D levels independent of renal production [80]. Finally, circulating levels of vitamin D do not determine the extent of its activity in target tissues, as the effects of vitamin D are ultimately transduced through VDR signalling, whose activity can be modified independent of vitamin D levels [81]. Given the complex nature of its metabolism and signalling, referring to systemic vitamin D levels alone may therefore be insufficient to fully understand its physiological impact, particularly under disease conditions. 

## 5. Current Understanding of the Mechanisms behind Vitamin D in Relation to VC

The impact of vitamin D on VC has yet to be fully elucidated and remains controversial to this day. While a large number of studies suggest that vitamin D excess (i.e., hypervitaminosis D) is associated with extensive calcification, others report that deficiency also promotes calcification, with long-term supplementation providing protective effects. Current evidence from experimental studies suggests a biphasic response of vitamin D activity, with either excess or deficient levels of vitamin D potentially leading to deleterious calcification outcomes [82,83].

### 5.1. Hypervitaminosis D and VC

Induction of calcification through hypervitaminosis with vitamin D has been demonstrated and well characterised in multiple animal models, including mice, rats, goats and pigs (see Table 1). Treatment of rats with sublethal doses (7.5 mg/kg) of vitamin D plus nicotine produces a lasting 10–40 fold increase in aortic calcium content, resulting in the calcification and destruction of medial elastic fibres, subsequently leading to arterial stiffness [84]. In goats and pigs, dietary supplementation of vitamin D promotes the development of aortic and coronary calcified lesions in association with elevated serum calcium and cholesterol levels [85,86]. Vitamin D induced calcification in mice is currently considered to be one of the more robust models of calcification, in which single doses of 500,000 IU/kg/day can produce severe aortic medial calcification after just 7 days following 3 consecutive days of initial treatment [87]. Interestingly, a recent study produced a variant of this model in which mice initially treated with a lower dose (100,000 IU/kg/day) for 7 consecutive days developed moderate aortic calcification outcomes at 28 days (unpublished). In addition to precursor forms of vitamin D, such as D2 and D3, dosing of its active metabolite, calcitriol, also produces diffuse and widespread soft tissue calcification that has been demonstrated in a time-dependent manner in rats [88]. Despite the number of in vivo models, evidence to explain the clear mechanisms of action by which excess exogenous vitamin D promotes calcification is still lacking.

Animal studies consistently observe that vitamin D induced calcification is concomitant with increased serum calcium, phosphate and calcium-phosphate product, which emphasises the idea that physiologically, vitamin D promotes calcification through stimulating free calcium and phosphate levels, which as previously discussed, lead to vascular osteogenesis and mineralisation. On the other hand, calcitriol has been shown to directly induce mineralisation of bovine VSMCs via the VDR in vitro in a dose-dependent manner [89]. Such VDR-mediated mineralisation is associated with increases in alkaline phosphatase activity and receptor activator of nuclear factor kappa-B ligand (RANKL)/osteoprotegerin (OPG) ratios, and decreases in PTH-related peptide expression levels [89,90]. Other studies have also suggested that vitamin D induction stimulates the expression of metalloproteinases (MMP’s), which are known calcification promoters. Increased MMP-9 expression was detected within the chondro-osseous junction of tibias of mice treated with calcitriol in comparison with controls [91]. A major limitation which has impeded further clarification of how vitamin D directly facilitates calcification is that effects from vitamin D shown in vitro are rarely confirmed using in vivo models, as vitamin D-induced calcification in vivo is frequently confounded by elevated calcium and phosphate levels. Given the current lack of consistent evidence, more studies are required to explore other mechanisms by which excessive vitamin D promotes calcification. 

### 5.2. Hypovitaminosis D and VC

Several studies have also challenged the view of vitamin D inducing and supporting calcification, with evidence that vitamin D deficiency, or hypovitaminosis D, is actually a stimulus to deleterious calcification outcomes. In uremic rat models, medial calcification in the aorta and ectopic calcification within soft tissue have been detected despite low levels of serum calcium and calcitriol, and instead have been associated with extremely high levels of PTH and inorganic phosphate [95]. Inadequate vitamin D intake has also been shown to enhance aortic calcification in wild-type and low-density lipoprotein receptor knockout (LDLR^−/−^) mice respectively [96,97]. Unfortunately, similar to hypervitaminosis, the mechanisms of action by which vitamin D deficiency promotes calcification development have yet to be fully elucidated. One of the major insights which addresses the potential protective role of vitamin D during calcification are studies concerning inflammation-dependent calcification. Clinical data and animal models have found an association between arterial calcification and increased levels of pro-inflammatory factors such as tumour necrosis factor alpha (TNF-α), interleukin-1 beta (IL-1β), interleukin-6 (IL-6) and the Msx2-Wnt signalling pathway [102,103]. Inflammation has also been documented to contribute to vascular cell mineralisation through stimulating mineral resorption and osteoclastic activity [26,104]. Physiological levels of vitamin D are capable of inhibiting calcification through modulating inflammation, with vitamin D deficiency leading to pro-inflammatory activity that subsequently drives calcification. Previous vitamin D deficiency studies involving LDLR knockout mice found increased TNF-α expression concomitant with the upregulation of osteogenic factors and aortic calcification in mice with inadequate vitamin D intake [96,97]. In an in vitro system of human VSMCs, high culture media phosphate concentration enhanced calcification through the induction of TNF-α expression [93]. The authors furthermore demonstrated that supplementation with calcitriol or the vitamin D analogue maxacalcitol reduced TNF-α levels in accordance with suppressed VSMC mineralisation. The mechanism for vitamin D deficiency in promoting inflammation-driven calcification may be explained by the impact of pro-inflammatory factors on the endothelium, which mediates endothelial stress and dysfunction that can then become the stimulus for calcification [105]. Indeed, vitamin D plays an important role in maintaining endothelial integrity, with deficiency linked to activation and dysfunction through the upregulation of inflammation, as well as oxidative stress and cell adhesion [106]. Interestingly, the primary endothelial stress mechanism currently known for calcification, which concerns the stimulation of the EMT process and the expression of BMPs and MGPs, has yet to be linked to vitamin D deficiency. Further studies into this area will elucidate speculations on not only vitamin D deficiency, but also the relevance of the endothelium on calcification development.

In addition to evidence suggesting that inflammation and endothelial dysfunction may be the primary mechanisms by which vitamin D deficiency promotes calcification, reports that vitamin D sterols can downregulate the RUNX2 pathway suggest its potential to directly inhibit the osteogenic differentiation process of VSMCs [94]. Finally, calcitriol and other vitamin D analogues can reduce the expression of the calcification promoters MMP-2, MMP-9 and vascular endothelial growth factor [107,108], although these findings do conflict with those previously discussed with regards to vitamin D excess supporting the expression of these calcification promoters. Some authors addressed these contradictory findings by emphasising that the effect of vitamin D on calcification regulators is transient, whereby normal levels of vitamin D regulate the expression of calcification promoters, while excess levels stimulate their expression [109]. Generally, many aspects of the anti-calcifying properties of vitamin D remain controversial due to the lack of clarification on its overall regulatory function on the risk factors for calcification, including mineral metabolism, inflammation, and molecular regulators of calcification.

### 5.3. Vitamin D Metabolism and Signalling: The Key to the Biphasic Response

While there is considerable evidence supporting that both vitamin D excess and deficiency promote calcification, the important question still remains as to how vitamin D is able to elicit such a biphasic response to ultimately induce calcification outcomes [110]. Indeed this is particularly difficult to clarify given how unpredictable it is to determine the net impact of perturbed vitamin D levels on physiological systems. Evidence provided by recent studies into this phenomenon suggest that rather than focusing on aberrant levels of the hormone itself, the impact of vitamin D metabolism in terms of synthesis and degradation, and its signalling within target tissues should also be considered [90]. Although this concept has yet to be directly examined, it may hold significant clinical implications as physiologically vitamin D is readily metabolised at multiple stages, with additional regulatory mechanisms modulating the production of metabolites and signalling at each stage. Furthermore, this concept is reflected by many of the hypervitaminosis and hypovitaminosis studies, the majority of which have shown the impact of vitamin D on calcification using different vitamin D derivatives. 

Ellam et al. provided critical insights into this concept by demonstrating the biphasic properties of vitamin D on VC development using an apolipoprotein E knockout mouse model [83]. While mice fed with a diet deficient in calcifediol (25-hydroxyvitamin D) developed increased aortic calcification density, those treated with paricalcitol, an analogue of calcitriol (1,25-dihydroxyvitamin D3), also developed severe calcification. Interestingly, calcification outcomes were associated with alterations in plasma calcium and phosphate levels from paricalcitol-treated mice, but not from those that were given the calcifediol-deficient diet. Furthermore, replenishment of calcifediol levels in calcifediol-deficient mice reduced the extent of calcification, but did not induce any changes in systemic calcium and phosphate levels [83]. Taken together, these results suggested differential functions of vitamin D derivatives on calcification development, in which the active vitamin D metabolite is able to induce calcification through the stimulation of calcium and phosphate, while its precursor form is able to provide protection against calcification instead via calcium and phosphate-independent mechanisms. Therefore, it is possible that other vitamin D metabolites and regulatory components have differential calcifying effects by acting through different mechanisms of action. Torremade et al. reinforced this idea by demonstrating pro-calcifying functions from 1α-hydroxylase using the uremia-induced model of VC. The authors were able to elegantly show that increased arterial 1α-hydroxylase activity contributes to progressive vascular osteogenesis and calcification in vitro and in vivo without changes in precursor vitamin D levels, reinforcing the idea that local regulation of vitamin D metabolism in vascular tissue can modify calcification outcomes to a similar degree as altered systemic vitamin D levels [99]. 

**Figure 1:**
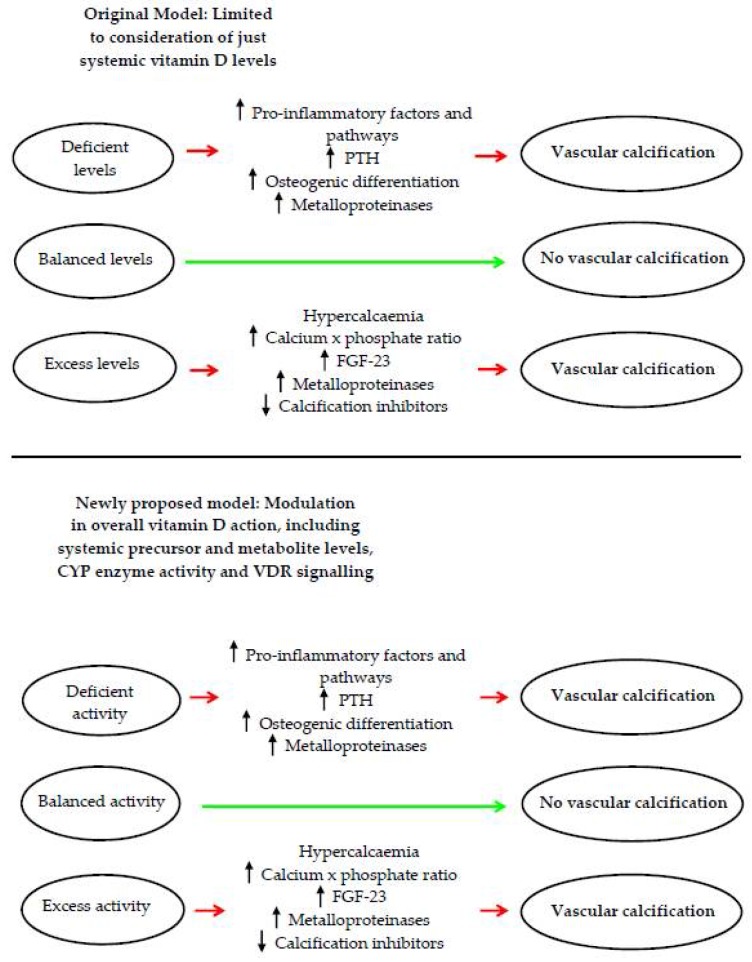
Schematic comparing the original and newly proposed models behind vitamin D’s biphasic response on vascular calcification. (Top panel): Original model concerning that perturbation of systemic vitamin D levels determine calcification outcomes; (Bottom panel): Newly proposed model that perturbation of overall vitamin D activity, comprising systemic levels, as well as vitamin D turnover and VDR signalling activity, collectively contribute to VC. FGF-23: fibroblast growth factor 23; PTH: parathyroid hormone.

Finally, the vitamin D receptor (VDR), which is the actual molecular mediator for active vitamin D signalling, has also been reported to elicit profound effects on VC. Both stimulation and ablation of VDR signalling promotes different types of calcification, such as arterial atherosclerotic calcification and medial calcification, indicating that VDR signalling itself impacts on VC by regulating gene expression programs within target tissues [88,97,100,101]. While the majority of the findings argue that moderate VDR signalling is necessary to provide overall protection against calcification, the exact mechanism of action of how VDR signalling impacts calcification-related events in the vasculature still needs to be determined. Collectively, these studies indicate that the biphasic response of vitamin D towards calcification may not be solely associated with its systemic levels, but a combination of its turnover and signalling activities in addition to its local tissue bioavailability, which will have cumulative effects that may be ultimately pro- or anti-calcifying (Figure 1). Therefore measurement of additional components, such as the levels of vitamin D metabolites, the activity of its metabolic enzymes and the level of signalling in target tissues, rather than vitamin D levels per se may provide a better assessment of how excess or deficiency both influence VC pathogenesis. 

## 6. Clinical Implications of Vitamin D in VC

The association between vitamin D and cardiovascular mortality is well established. Deficient vitamin D levels, in the form of calcifediol, has been linked with increased cardiovascular and non-cardiovascular mortality in a number of large-scale clinical and epidemiological studies [76]. The predominant finding from these studies is a non-linear, inverse relationship between serum calcifediol concentration in patients and cardiovascular risk, with levels below 24 ng/mL in particular exhibiting the highest relative risk scores [111,112,113,114,115,116]. In one prospective study, serum calcifediol concentrations of less than 20 ng/mL in pre-hospitalised patients were associated with increased odds of 90-day mortality [117]. More significant correlations between vitamin D deficiency and cardiovascular mortality have also been reported from older populations [116,118], and in female cohorts [119]. In the majority of these association studies, composite cardiovascular outcomes, including VC, were major contributors to increased patient mortality, and were also correlated with deficient vitamin D levels. Although these studies suggest that vitamin D deficiency increases cardiovascular risk by promoting VC, clinical evidence for worsening VC outcomes in response to deficient vitamin D levels is still controversial. Several retrospective studies have demonstrated improvements to cardiovascular mortality in patients through supplementation of vitamin D metabolites, analogues and receptor activators, however they fail to address how treatment of vitamin D activity has altered their VC outcomes [120,121,122,123,124,125]. Furthermore, the mechanisms shown in vitro and in vivo for vitamin D deficiency and induction of calcification in relation to inflammatory markers and calcification promoters have yet to be established in clinical cases. Therefore, justification for the clinical use of supplementation in ameliorating calcification progression still needs to be validated. 

On the other hand, while the focus of the impact of vitamin D on calcification has been primarily on vitamin D deficiency, limited as they may be, studies which have independently examined the relationship between hypervitaminosis D and calcification in patients are also scarce. This may be largely due to the fact that hypervitaminosis D is rarely seen in human populations, and also that the induction of vitamin D toxicity in patients is very impractical, given that homeostasis of vitamin D is tightly regulated under physiological conditions, and that over-supplementation may be ethically impractical [126]. Furthermore, it should be noted again that circulating levels of active vitamin D does not truly reflect loading or supplementation of precursor vitamin D due to the function of vitamin D metabolic enzymes, such as 1α-hydroxylase and 25-hydroxylase [127]. Based on these postulates, the clinical impact of vitamin D excess in promoting calcification may be less important than that of deficiency, but should not be disregarded. 

Interestingly, one clinical study performed on a paediatric cohort highlighted the biphasic response of vitamin D with regards to calcification, in which patients with low or high levels of vitamin D exhibited significantly greater carotid intima-media thickness and calcification scores than those with normal levels [92]. Furthermore, calcification outcomes were associated with higher levels of high-sensitivity C-reactive protein indicating systemic inflammation within the low level group, while those of the high level group were linked with deregulated calcium/phosphate metabolism. These clinical findings reinforce the idea that physiological levels of vitamin D are necessary to avoid the activation of pro-calcifying mechanisms, therefore confer calcification resistance, while perturbation of its homeostasis through excess supplementation or depletion ultimately triggers mechanisms that lead to pathogenesis. Unfortunately, no clinical studies have been performed examining the entire spectrum of vitamin D metabolic and signalling activity, in addition to systemic levels, on calcification development, which so far has only been alluded to by experimental evidence. With the range of clinical findings on vitamin D and VC still requiring further validation, current and future studies should account for more parameters of vitamin D activity during VC pathophysiology, which may ultimately justify the therapeutic or detrimental potential of vitamin D for VC. 

## 7. Conclusions

VC is a degenerative vascular disease which typically affects major branches of the arterial tree, and independently contributes to deranged cardiovascular outcomes and increased mortality. The majority of calcified tissues are characterised by not only inert mineral deposits, but also dynamic bone elements indicating that it is an active pathogenic process which mimics some features of ossification. While traditional atherogenic stimuli have been considered to contribute to VC pathogenesis, recent findings indicate more important roles for non-traditional risk factors, such as hormonal perturbations and disturbed mineral metabolism in accounting for the aberrant VC clinical outcomes related to severe cardiovascular events. In particular, deregulated calcium and vitamin D levels facilitate calcification through stimulating both VSMC osteogenic differentiation and mineralisation. Current understanding of vitamin D’s involvement during VC is limited, and primarily relies on evidence provided from experimental studies. Both hypervitaminosis and hypovitaminosis D can contribute to the development of VC via multiple complex mechanisms, indicating the biphasic impact of vitamin D on the vasculature. Although the specific molecular mechanism remains to be fully clarified, understanding how modulations in systemic vitamin D levels changes its metabolic and signalling activity during VC development may offer more insights into explaining the biphasic response. On the other hand, clinical evidence of the biphasic impact of vitamin D on VC in patients is limited, with current guidelines suggesting that tightly regulated levels of vitamin D are ultimately necessary to prevent VC. Further studies are required to elucidate the underlying mechanisms of how the vitamin D hormone, its metabolism and signalling via the VDR interacts with calcification processes and risk factors to clarify therapeutic or detrimental impact in disease pathogenesis and development. 

## Figures and Tables

**Table 1:** Published studies on the impact of vitamin D on vascular calcification (VC).

Aspect of Vitamin D Examined	Study Model	Proposed Mechanisms for VC	Reference No.
Hypervitaminosis	In vitro	Modulation of alkaline phosphatase activity, RANKL/OPG ratio and PTH-related peptide expression	[89]
	Animal	Increase of free calcium levels and osteogenic factors. Direct mineralisation of VSMCs.	[83,85,86,87,88,91]
	Human	Deregulated calcium and phosphate metabolism	[92]
Hypovitaminosis	In vitro	Stimulation of the expression of TNF-α and osteoblast differentiation factors.	[93,94]
	Animal	Low free levels of calcium and calcitriol in association with high levels of PTH and inorganic phosphate. Stimulation of the expression of TNF-α and osteoblast differentiation factors. Induced expression of osteogenic factors independent of calcium and phosphate levels	[83,95,96,97]
	Human	Direct regulation of osteoblast function by calcitriol dependent on serum levels. Increase in the levels of high sensitivity C-reactive protein	[92,98]
Vitamin D metabolism	In vitro	VSMC osteogenesis and calcification induced by increased 1-α hydroxylase expression independent of vitamin D levels	[99]
	Animal	Promotion and suppression of aortic calcification by different vitamin D derivatives	[100,101]
VDR signalling	In vitro	Suppression of the expression of osteogenic factors through calcitriol-mediated VDR activation and subsequent signalling	[94]
	Animal	Stimulation of the expression of osteogenic factors through VDR signalling. Promotion and suppression of aortic calcification by different VDR activators	[88,100,101]

RANKL—receptor activator of nuclear factor kappa-B ligand; OPG—osteoprotegerin; PTH—parathyroid hormone; VSMC—vascular smooth muscle cell; TNF-α—tumour necrosis factor alpha; VDR—vitamin D receptor.
